# Looking Back to Move Forward: Reflections on the Strengths and Challenges of the COVID-19 UK Mental Health Research Response

**DOI:** 10.3389/fpsyt.2021.622562

**Published:** 2021-04-08

**Authors:** Ola Demkowicz, Margarita Panayiotou, Sam Parsons, Amy Feltham, Louise Arseneault, Beth Ingram, Praveetha Patalay, Dawn Edge, Matthias Pierce, Cathy Creswell, Christina Victor, Rory C. O'Connor, Pamela Qualter

**Affiliations:** ^1^Manchester Institute of Education, The University of Manchester, Manchester, United Kingdom; ^2^Department of Experimental Psychology, University of Oxford, Oxford, United Kingdom; ^3^Independent Researcher, London, United Kingdom; ^4^King's College London, Institute of Psychiatry, Psychology and Neuroscience, London, United Kingdom; ^5^Hearts and Minds, London, United Kingdom; ^6^Centre for Longitudinal Studies and Medical Research Council Unit for Lifelong Health and Ageing, University College London, London, United Kingdom; ^7^Division of Psychology and Mental Health, The University of Manchester, Manchester, United Kingdom; ^8^Greater Manchester Mental Health Trust (GMMH) National Health Service Trust, Manchester, United Kingdom; ^9^The Manchester Centre for Women's Mental Health, The University of Manchester, Manchester, United Kingdom; ^10^Department of Psychiatry, University of Oxford, Oxford, United Kingdom; ^11^College of Health and Life Sciences, Brunel University London, London, United Kingdom; ^12^Institute of Health and Wellbeing, University of Glasgow, Glasgow, United Kingdom

**Keywords:** COVID-19 pandemic, mental health research, open science, coproduction, robust methods, workforce inequality

## Abstract

In the face of the COVID-19 pandemic, the swift response of mental health research funders and institutions, service providers, and academics enabled progress toward understanding the mental health consequences. Nevertheless, there remains an urgent need to understand the true extent of the short- and long-term effects of the COVID-19 pandemic on mental health, necessitating ongoing research. Although the speed with which mental health researchers have mobilized to respond to the pandemic so far is to be commended, there are valid concerns as to whether speed may have compromised the quality of our work. As the pandemic continues to evolve, we must take time to reflect on our initial research response and collectively consider how we can use this to strengthen ensuing COVID-19 mental health research and our response to future crises. Here, we offer our reflections as members of the UK mental health research community to discuss the continuing progress and persisting challenges of our COVID-19 response, which we hope can encourage reflection and discussion among the wider research community. We conclude that (1) Fragmentation in our infrastructure has challenged the efficient, effective and equitable deployment of resources, (2) In responding quickly, we may have overlooked the role of experts by experience, (3) Robust and open methods may have been compromised by speedy responses, and (4) This pandemic may exacerbate existing issues of inequality in our workforce.

As the coronavirus disease 2019 (COVID-19) pandemic has unfolded, the mental health research community has been keen to contribute to the rapid research response. Researchers have swiftly initiated and undertaken a wealth of research projects, facilitated to an extent through existing research infrastructure^1^.

We do not yet know, however, the extent to which these efforts have achieved the desired outcomes, including advancing understanding of the impact of COVID-19 and the ongoing social distancing and national/regional lockdowns measures on mental health. Since this rapid response will continue, it is important we reflect on initial activity to consider how we can apply any learning moving forward. Here, we offer reflections on the initial COVID-19 mental health research response, with an emphasis on the United Kingdom (UK; March–October 2020). These capture the perspectives of the authors. We are a group of UK mental health researchers (including academics involved in the COVID-19 research response), experts by experience, and academics overseeing relevant networks and journals. We set out to focus on the processes through which initial COVID-19 research activity has been undertaken, using that to stimulate discussion and reflection among the mental health research community as we continue and plan our next steps in responding to the pandemic and beyond. Unlike the work by Holmes, O'Connor and colleagues (1) and O'Connor and colleagues (2), the current perspectives piece does not focus on *what* should be prioritized; rather, we consider the mental health response to COVID-19 so far and offer suggestions on *how* what we have learned can be applied to ongoing COVID-19 mental health research, future emergency situations, and general research practice. Here, we offer discussion of four key reflections. Boxes 1 to 4, offered throughout the text, summarise key challenges and considerations moving forward.

Box 1 **Reflection 1 summary and key considerations moving forward**

**Systems have been streamlined to allow for a speedy response**
  • Funders and HEIs must learn from COVID-19 research streamlining, working to create sustainable models with more straightforward and timely application processes.
  **Our research response shows fragmentation and an overlapping emphasis on specific priorities**
  • Efforts to further develop and expand cross-institutional and interdisciplinary networks should be undertaken to support greater collaboration and more efficient resource deployment.
  • There is a need for continued effort and research in finding ways to bring our community together to create a more collaborative approach (e.g., funders considering how their calls can directly encourage cross-institutional collaboration).
  • Registries can aid coordination of activity, but these too ought to be carefully coordinated to avoid duplication.
  **Our infrastructure has not been built for emergencies**
  • Leaders and coordinators should further develop our infrastructure to better facilitate research responses to future emergencies, particularly given calls to do so over a decade ago (3).

Box 2 **Reflection 2 summary and key considerations moving forward**

**There are some concerns about the extent of co-production in COVID-19 research, likely due to the speedy response and the challenges brought by the pandemic**
  • Researchers must consider ways in which they can engage individuals with lived experience who might be more difficult to reach remotely [see for example tips on how to co-produce during COVID-19; (4)].
  • Funders must ensure that more small-scale funding is available to *specifically* support the effort and costs of co-production: during the development of the research proposals, and to support training around co-production, especially for research teams that lack the initial funds or the infrastructure to achieve this.
  • Researchers should be aware of the challenges of co-production that may have been magnified by the pandemic (e.g., the time and effort required to build these relationships) and plan for a more flexible collaboration with co-production and PPIE representatives.

Box 3 **Reflection 3 summary and key considerations moving forward**

**Lived experience has been overlooked due to limited qualitative research**
  • Future work should consider adopting mixed methods. Where this is hindered by lack of expertise, the research team should seek to initiate collaborative, interdisciplinary research with experts of this method.
  **New COVID-19 studies have relied heavily on less reliable sampling methods (non-probability samples through online surveys)**
  • Future COVID-19 work should consider the use of random sampling methods to reduce bias.
  • Researchers interested in exploring COVID-specific effects should consider using existing cohort studies which include pre-COVID data.
  **Much of the current work relies on general populations**.
  • Future work should focus on establishing the long-term experiences of specific marginalized populations.
  **Some of the existing and new COVID-specific measures used in COVID-19 mental health research lack sufficient validation and measurement invariance**
  • Researchers must provide transparent information about measurement practices (how they chose, quantified, validated, and/or modified a measure) which will enable reproducibility and accurate interpretations of findings
  • Researchers must seek to establish measurement invariance in their own sample, before meaningful comparisons can be made.
  **Paper-and-pencil questionnaires and face-to-face interviews moved to a web or telephone mode**.
  • Researchers must consider possible mode effects within analysis, interpretation, and reporting. Though a shift in remote data collection was possible for psychosocial assessment, this has been more challenging for biological data, especially in populations deemed more vulnerable.
  **In some cases sub-optimal statistical methods have been employed**
  • Where researchers are interested in bidirectional associations over time, they must ensure that up-to-date robust analytic methods (i.e., random-intercept panel model) are employed to ensure the accuracy of results.
  • Collider, confounders and mediators must considered in analysis for accurate causal effects. Expert knowledge can ensure that the appropriate variables are considered in a model.
  • Researchers designing new studies must ensure that appropriate confounders (e.g., biological, social) are assessed, though we note that difficulty in collecting biomedical data is likely. This requires interdisciplinary work and collaborations between different sectors and disciplines.
  **Funders, researchers, and the public placed a greater importance on open science practices, than usual**.
  • There has been open sharing of research protocols and questionnaires between research teams, and an increased publication of preprints, in an effort to allow for rapid dissemination.
  • However, in comparison, pre-registration of study designs and statistical analysis plans has been limited. Researchers should thus ensure the pre-registration of studies prior to data analysis (e.g., in platforms such as the Open Science Framework).
  • For future work, researchers must also ensure they actively ask for participants' permission to safely deposit their data for re-share and re-use. During this process, transparency around sharing data, protecting privacy, and ensuring anonymity of sensitive mental health data must be ensured. The UK Data Service is one example of a platform for securely depositing, managing, and sharing research data [e.g., see the Understanding Society COVID-19 data; (5)].

Box 4 **Reflection 4 summary and key considerations moving forward**

**Researchers in precarious contracts are now in vulnerable positions**
  • We urge urgent examination and action within the UK research infrastructure to create better support and greater stability to those on casual or precarious contracts.
  • HEIs should invest in decasualising their workforces, both to protect current researchers and to ensure we are able to recruit and retain new researchers
  • Funding bodies should consider their role in facilitating better employment, such as creating conditions within grants to utilize open-ended contracts.
  **The pandemic has created unequal consequences across our workforce, with some groups likely to be hit harder than others**
  • As precarious contracts are disproportionately held by women and UK ethnic minorities HEIs and funders should urgently consider the inequalities being exacerbated as a result of COVID-19.
  • Our infrastructure must be developed to ensure support and opportunities for women and UK ethnic minorities given their over-representation in casual employment.
  • HEIs, funders, and networks should be considerate of the barriers faced by some (e.g., parents and those with mental and physical health issues) and take steps to limit the consequences of lowered publication and funding during 2020.

## Reflection 1: Fragmentation in our Infrastructure has Challenged the Efficient and Equitable Deployment of Resources

The pandemic has affected all spheres of life and highlighted the value of work across disciplines and sectors. We start this section by acknowledging that networks and funding bodies facilitating interdisciplinary work have come into their own, launching rapid funding opportunities because of their intrinsic flexibility [e.g., see examples of COVID-19 research supported by the UKRI Mental Health Networks; (6)]. Those calls were met by extraordinary organizational efforts from higher education institution (HEI) research services that expedited processes within institutional ethics review committees to allow for a rapid response. Notably, funding bodies also streamlined systems, with briefer application processes and swift decisions. That offers a critical lesson: academics typically invest substantial time and effort on funding applications, but given the over 80% rejection rate (7, 8), this is wasteful and contributes to burnout, lowered productivity, and poor wellbeing (9). During COVID-19, funders have demonstrated that streamlined systems can reduce burden for everyone involved, apparently without compromising their decision-making capacity. However, a more detailed analysis is required to determine whether certain research streams, such as mental health interventions were disadvantaged by this approach. Nonetheless, we urge funders and HEIs to develop sustainable models with more straightforward and timely application processes, while still preserving quality and rigor in the review process.

Yet, the pandemic has also highlighted the weaknesses of our research infrastructure. We specifically note fragmentation in our response, coupled with an over-emphasis on specific priorities. The prioritization and coordination framework developed by Holmes, O'Connor and colleagues (1) has offered a decisive starting point from which to coordinate and deploy effort and resources, and we acknowledge that there has been important national and international collaborative effort. However, our initial research response was relatively fragmented, evidenced by the sheer volume of research studies with overlapping designs; for instance, registries show many studies capturing quantitative data around depression, anxiety, and loneliness among those aged 16 years and older (10, 11). Within a more advanced infrastructure, with expansive cross-institutional networks and fewer siloed research approaches, we may have done better: we could have identified possible synergies and initiated collaborations where priorities overlapped, producing a smaller number of large-scale studies but with larger teams and more concentrated resource deployment. Whether academic silos should be brought down in their entirety is a complex issue, not just for mental health research (12). What we suggest here is that we must find ways in which we can bridge these, for moments, such as this, where collective effort may have been more effective. Of course, we are aware of the practical and motivational challenges behind collaborations between research teams, especially when they must compete for funds and academic publications. We hope that in the future there is greater effort and research in understanding how to bring colleagues together. Given the competitive funding landscape of mental health research, funders can make a significant first step forward by offering, for instance, large-scale, yet equitable, funding schemes with cross-institutional collaboration in their core.

The fragmented research response appears to reflect and perhaps exacerbate an emphasis on short-term impact and rapid response as well as general population investigation; though such efforts are critical (1, 3), we must avoid prioritizing them over long-term efforts and exploration of impact for those at risk of long-term and acute effects (e.g., UK ethnic minorities, children and young people, and those with existing mental health difficulties). Fragmentation has also placed unnecessary burden on members of the public who are keen to engage in research, led to services being bombarded with adverts to share with users, and may have resulted in overlapping participants across samples, complicating meta-analytic efforts. It also revealed that interdisciplinary work has, thus far, been limited: there has been a greater emphasis on self-report psychosocial data, despite, for example, the benefit biological data could offer [e.g., understanding how social and biomedical characteristics may explain the COVID-19 burden among older people; (13)]. Although this fragmentation is not unique to mental health research [e.g., COVID-19 medical research; (14)], it unmasks ongoing weaknesses in our infrastructure and highlights that this infrastructure has not been built for emergencies, despite calls to do so over a decade ago (3). Leadership and coordination are needed going forward. Some good examples so far include coordination activities, such as the COVID-MINDS Network (10), the NIHR COVID-19 and Mental Health Studies Register (11), and the COVID-19 Suicide Prevention Research Collaboration (15), although we also warn against the use of multiple fragmented registries because that risks further duplication and inefficiency. Ultimately, if we are to deploy large amounts of time, energy, and funding to facilitate a rapid response, we must ensure it is done strategically and with coordination. This is something funders should have a more active role on. We, therefore, recommend that our existing infrastructure (e.g., existing networks and cohorts) is enriched, with an emphasis on collaboration, interdisciplinarity, and cross-sector partnerships. It is also important that we build longer-term mechanisms that allow the mental health research community to act proactively in the face of emerging crises.

## Reflection 2: In Responding Quickly, We May Have Overlooked the Role of Experts by Experience

The need for fast, reactive research has both affected and reflected the state of co-production in mental health research. Research *for* people struggling with their mental health in the COVID-19 crisis is well-meaning. It misses out, however, on the richness and relevance co-produced research can bring, wherein people with lived experience are essential, equal partners, and the aim is to do things *with* or *by* them (16, 17). Co-production and patient and public involvement and engagement (PPIE) can strengthen research in various ways. It empowers groups that may be seldom heard, thus challenging power dynamics within research and allowing for more relevant and meaningful findings (16, 17).

We note that several mental health projects have incorporated co-production and PPEI to good effect in responding to the pandemic. These include, for instance, a qualitative project from Gillard et al. (18) wherein the research team included individuals with lived experience of mental health problems and a mixed method study exploring mental health and coping strategies among young people from Dewa et al. (19) that describes itself as young-person led. Resultantly, these and other similar projects have been able to prioritize and focus effectively and produce highly relevant and nuanced outputs with clear practical implications.

Though such good examples exist, it seems that in many cases co-production and PPIE may have unfortunately fallen by the wayside in the haste of our response. Already, research ethics committees report that inclusion of co-production and PPIE appears to have been substantially lower in COVID-19 rapid health research, relative to “normal” research (20). However, that does not need to be the case moving forward. The existence of established networks has supported aspects of practice, including the PPIE and co-production within COVID-19 research (e.g., UKRI networks and organizations, such as The McPin Foundation). Thus, in organizations and networks where people with lived experience are already viewed as equal partners, (21), and where resources are available to support engagement, good quality research can still turn around relatively quickly, with the capacity for stronger, more relevant results. Co-production should not be viewed as “another thing to get done,” but as a way to accomplish relevant, meaningful research (22). Now more than ever, mental health research must be driven by all who are likely to be impacted by the pandemic (23). Indeed, the pandemic has the potential to allow for the possibility for greater inclusivity and broader relevance, and can create a space for individuals with lived experience to be involved in (24). While we are not able to directly assess the extent to which lived experience within COVID-19 work has been incorporated or overlooked, others have noted concerns about lowered attention to this (25). Moving forward we urge research groups to find, recruit, and train [e.g., see Campaign Bootcamp; (26)] people with direct lived experience relevant to their research aims, to play an active role in all stages of research, both during the pandemic and beyond.

When including individuals with lived experience in future research, researchers must also consider practical matters, and especially ongoing challenges that have arisen due to the pandemic. For instance, the new normal of working from home and remote engagement methods may facilitate greater engagement with individuals who are often overlooked within co-produced research, for instance because they cannot easily leave home. This demonstrates that so-called “hard to reach” populations can, in fact, be reached with appropriate adaptations. Still, we must not forget those who may struggle to engage virtually, for instance inpatients, frontline workers, or those with unstable living conditions (27). Given that co-production with those populations often relies on face-to-face meetings, researchers must consider other ways to involve all individuals with lived experience, by ensuring, for instance that those without internet access have instead access to a free phone number (28, 29). Researchers must be aware of other practical issues, such as the costs associated with co-producing research and the availability of funds to accommodate this. Indeed, researchers without funding or established networks were less able to meaningfully incorporate such perspectives at speed (30). This highlights a need to develop suitable PPIE and co-production groups within our research infrastructure, and to offer rapid, light touch funding streams to support such efforts, such as the small-scale funding provided by NIHR Research Design Services (4). Finally, researchers should be mindful of the time and effort needed in building relationships with individuals with lived experience (31) and plan for a flexible partnership, as this may be harder to sustain virtually, especially as co-production and PPIE representatives may have additional responsibilities during this time (32). As restrictions lift, mental health research should embrace the ability, but also the responsibility, to work with people with lived experience of COVID-19 and mental health difficulties.

## Reflection 3: Robust and Open Methods May Have Been Compromised by Speedy Responses

The thirst for information can threaten the credibility of our work. Findings based on inappropriate methods, samples, and untenable evidence risk misinforming the public and, crucially, policymakers. It is our responsibility to be open and transparent about the methodological strengths and pitfalls of our work, and we argue this should be a strong driver for good research and open science practices. Here we reflect on how these have been applied in the COVID-19 effort thus far.

## Limited Qualitative Inquiry

Notably, the initial rapid research response to the pandemic was overwhelmingly quantitative. At the time of writing, just one in five of the studies registered in the NIHR COVID-19 and Mental Health Studies Register reported use of qualitative and/or mixed methods (11). Encouragingly, these predominantly explore experiences among specific subgroups, such as adolescents (33), mental health service users (34), and foster carers and children in care (35), facilitating deeper understanding of differential impact. However, the limited number of qualitative studies raises concerns. Despite growing recognition that qualitative inquiry can uniquely inform policy and mental health services (36, 37), statistical analyses are overwhelmingly favored over lived experience and many journals treat qualitative studies as low priority or directly discourage submissions (37–39). We are currently faced with complex circumstances across the globe, with stark disparities in illness and morbidity rates, loss of mourning and bereavement rituals, widespread economic loss and anxiety, repeated shifts in restrictions, and school closures, to name a few. We question how effectively we can understand statistical associations or translate knowledge into meaningful action without a deeper understanding of what those issues represent within people's lives. Valuing different methodological approaches and developing an integrated mixed methods evidence base in mental health is key for facilitating a nuanced and meaningful strategy to policy and practice (36, 37).

## Methodological Concerns

On the other hand, early efforts to quantify the impact of the pandemic on mental health relied on online surveys using convenience samples, often recruited via social media. Surveys tend, however, to under-sample groups (e.g., older people, homeless, men, minorities) and over-sample people engaged with the issue at hand. Additionally, from a theoretical perspective, it is not possible to correct for the sampling bias of non-probability sampling (as the probability that somebody is in the sample is unknown), and statistical tests on these samples require more complex modeling and stricter assumptions (40, 41). Therefore, while non-probability samples enable quick and cost-effective results, they provide less reliable prevalence estimates (42); drawing inferences from those is problematic, necessitating caution when interpreting and feeding COVID-19 findings into policy.

We recognize the important efforts of those involved in rapidly initiating longitudinal COVID-19 surveys (10); indeed, those studies will provide useful information about continuous experiences and needs of individuals during the pandemic. But, without appropriate pre-COVID-19 data these studies tell us little about whether changes in mental health are pandemic-specific. Existing cohorts can be valuable in addressing this (43), but many focus exclusively on general populations, highlighting an urgency to establish new research with marginalized populations, such as residents and staff of care homes.

Issues with measures used within COVID-19 research (e.g., GAD-7, UCLA, used in around 36% of the COVID-MINDS studies) must also be considered. First, researchers must ensure their chosen measures are valid, assessing what they are meant to be assessing. This is especially the case for measures that have been validated with convenience samples, such as the UCLA loneliness scale (44), COVID-specific measures that have been hastily developed but lack detailed validation (45), and, more generally, measures that have been developed without the input of the intended audience (46), as is often the case with children and young people (47). Now, more than ever, the validity of measures, especially across groups, must be considered, as COVID-19 has a disproportionate impact on the health of older individuals and UK-ethnic minorities (48, 49). Lockdown approaches have also varied across the world, immediately creating a scientific interest to consider age, cross-cultural, and cross-national differences. As simple as this sounds, often measures are non-invariant, assessing different things across samples and/or groups, as is the case, for instance, with GAD-7 (50). It is, therefore, urgent to establish measurement invariance, before meaningful comparisons can be drawn. Finally, we highlight potential comparability issues with the pre- and post-pandemic measurement of mental health. Paper-and-pencil questionnaires and face-to-face interviews moved to a web or telephone mode (51). That can affect the way in which individuals respond to the same questions, by giving different answers in different modes [also known as mode effects; (52)]. Researchers must consider that issue within analysis, interpretation, and reporting (53).

Robust methods can still prove insufficient if analyses are inappropriate. First, during the investigation of potential COVID-19 causal effects researchers need to carefully consider which variables to include in their models. The inclusion of a collider (i.e., a third variable caused by the independent and outcome variables) (54), and failure to consider key biological and psychosocial mediators or confounders (54, 55), can lead to spurious causal effects (though we note there may be challenges in collecting biological data at this time, particularly with high-risk groups). For instance, given the increased focus on loneliness during the pandemic, researchers assessing this should also consider social anxiety as a possible confounder given prior evidence (56, 57) and recent qualitative COVID-19 evidence (58). However, this has been generally absent from the current work. This is one of the areas where interdisciplinarity and cross-institutional collaboration would prove most useful, as some of these confounders can only be identified by expert knowledge (54). Second, given the wealth of longitudinal studies, we anticipate increased use of cross-lagged panel models. However, some of the COVID-19 studies are already employing the traditional panel model, which fails to consider “trait-like” differences (between-person effects), and, thus, does not represent true within-person relationships over time (59). We, thus, urge adopting an appropriate method (e.g., random-intercept panel models) to avoid inaccurate conclusions about causal processes.

## Open Science Practices

There has been widespread, publicly stated commitment to ensure COVID-19 related research and data are made available, including from funding bodies (e.g., UKRI), research journals, institutions (60), and researchers (10). Early on, researchers shared study protocols, questionnaires, and materials. Consequently, there may have been greater methodological consistency across ongoing research than usual, which might enable researchers to better address important questions and triangulate results across samples.

However, given that rapid dissemination is needed when research has vital policy implications and when communicating expert opinion to policymakers (61), COVID-19 mental health research is being conducted, and disseminated, at a rapid rate. At the time of writing, the COVID-MINDS network had indexed 225 published papers (10), and a search for “COVID AND mental AND health” found 726 preprints on medRxiv, and 128 preprints on PsyArxiv since 8th March 2020. Both preprints and journal articles can perform well as parallel means of communicating ongoing mental health research [see Fraser et al. (62) for an in-depth discussion], although sub-optimal methodological practices within COVID-19 research necessitate caution in basing policy decisions on preprints. For instance, despite the large number of COVID-19 preprints, we found only 93 preregistrations of analysis plans on the Open Science Framework using the above search term. Among other benefits, preregistration is useful for differentiating planned (confirmatory) from unplanned (exploratory) analyses (63). Open science practices have clearly not been abandoned, but it is possible that desire for more knowledge quickly meant open science was not applied with the same rigor. For instance, it is not yet clear the degree to which current COVID-19 studies have actively asked for participants' permission to safely deposit their data for re-share and re-use. Sharing research data and code plays an important role in increasing the impact of mental health research, maximizing the value of participant and patient contributions, reducing research waste, and may help treatment or service provision for others (64, 65).

It is encouraging that in the wake of COVID-19, mental health research has overall embraced the ethos of open science. It is crucial that researchers build on this, ensuring that, moving forward, this initial enthusiasm translates into new norms of openness and sharing.

## Reflection 4: This Pandemic May Exacerbate Existing Issues of Inequality in our Workforce

The pandemic has also highlighted ongoing issues of inequality, not just within the research response, but academia more widely. Academia holds inequality at the best of times, due to hierarchical structures and ongoing reliance on doctoral students and precarious roles held disproportionately by women and UK ethnic minorities (66). The pandemic has exacerbated that. Doctoral students and precarious staff have already begun experiencing impaired research, increased stress, and worry about future plans since the pandemic began (67–69). Many will be left with expired visas and no job prospects (70), in a system with limited opportunities for those without funding or not embedded in established networks. Others have faced intensified barriers and lowered research productivity, including parents with increased caregiving responsibilities, particularly mothers (71, 72) and those with existing mental and physical health issues. This may have long-term impact given that academic incentives reward publication and funding (72).

Ultimately, COVID-19 is likely to further widen inequalities within academia and potentially roll back progress that was being made in addressing structural inequalities. It is ironic that we are losing, and failing, to support a significant part of our workforce at a time of need for producing rapid mental health evidence (73). Even before the COVID-19 crisis hit, research showed that casual academic employment is a separate insecure secondary labor market within the academic workforce (74) as a result of universities relying on a business model that sees precarious academics, especially women, as non-citizens of the academy (75, 76). Failure to address such systemic issues may influence our ability to recruit and retain early career researchers (77), impacting our ability to deliver a mental health research agenda both in “normal” times and emergency situations. There is, therefore, an urgent need for a close examination of the UK research infrastructure and ways in which we can provide better support, and greater stability to researchers on casual or precarious contracts. These include some of the long-term recommendations by the University College Union, such as for research funders to make it a condition of grants to employ researchers on open-ended contracts, and universities to invest in the decasualisation of their workforces (75).

## Concluding Remarks

Swift efforts by funding institutions, HEIs, and mental health researchers has allowed important progress toward researching the COVID-19 consequences for mental health in the UK. However, there are valid concerns as to whether *speed* may have compromised elements of *quality*. We have reflected on and identified potential issues within the mental health research response thus far, which we note may have relevance for mental health research even in non-emergency contexts and indeed research within other fields. These include issues with fragmentation and duplication, the focus (e.g., unclear research questions), methods, co-production, and open science approaches adopted by COVID-19 mental health studies, and implications for equality in our workforce. It is difficult at this stage to comment on the implications those issues will have had for the quality of our work, but we note a need for caution and care when reviewing the current evidence, especially when this is used to drive policy.

The COVID-19 pandemic is evolving, necessitating flexibility and a need to continually review our research effort and apply learning moving forward (78). We note that the reflections presented here are a snapshot of our perspectives in late 2020, but continue to apply to more recent work, in which the same strengths and ongoing challenges appear present. Moving forward, research networks and organizations must make a conscious effort to minimize duplication and waste, facilitate collaborative and interdisciplinary work, and coordinate next steps, while identifying and retaining important advances. The mental health research community's response has highlighted how crucial the right infrastructure is in enabling rapid and efficient collaboration across disciplines and sectors, and with people with direct lived experience. So far, it is a good sign that UKRI funding programmes are currently continuing as before (79), the Wellcome Trust has confirmed their research support will continue for now (80), and NIHR has committed to funding research exploring the effects of COVID-19 beyond the acute phase (81). We have high hopes for other initiatives to develop interdisciplinary teams to address key mental health research challenges (82), and encourage funders to maintain mental health research. Major initiatives on mental health set by the UK government during this crisis [e.g., tackling loneliness; (83)] must not be short-lived, and appropriate long-term funding plans need to be considered even as the government comes under pressure to manage national debt and respond to shifts relating to Brexit.

As the situation continues to change, we must develop dynamic ways to review, respond, and share information openly. With appropriate funding in place, that could involve scoping exercises (e.g., rapid evidence reviews) to identify emergent trends, and, crucially, ongoing consultation to understand the needs of stakeholders. Whatever the approach, we must ensure we conduct robust and open research that builds a nuanced, meaningful, and robust evidence base to promote understanding of the mental health consequences of the pandemic. More widely, this is a critical opportunity to reflect on ongoing and new issues within our infrastructure and community, engage in collective discussion, and encourage action that can develop and strengthen our research field for the pandemic and beyond.

## Data Availability Statement

The original contributions presented in the study are included in the article/supplementary material, further inquiries can be directed to the corresponding authors.

## Author Contributions

OD and MPa initiated the manuscript, scoped the COVID-19 mental health databases, coordinated the reflections, and wrote the first draft. OD, MPa, SP, AF, PQ, and MPi led on writing the specific sections. PQ, LA, and CC scanned the current COVID-19 funding calls. All authors contributed to the writing of specific sections. All authors contributed intellectual content to the manuscript, edited first drafts, and read and approved the final manuscript.

## Conflict of Interest

OD is the Principal Investigator of the TELL Study (Teenagers' Experiences of Life in Lockdown; partially funded by The University of Manchester Economic and Social Research Council Impact Account fund). MPa is the Principal Investigator of the Covid-19, Social Media & Mental Health study (unfunded), and is involved in the Survey of Health and Wellbeing–Monitoring the Impact of COVID-19 (led by Dr. Michelle Lim, Iverson Health Innovation Research Institute, Swinburne University). SP is working on the “Oxford Achieving Resilience during COVID study” (Oxford ARC study); this project is funded by the ESRC [ES/R004285/1]. PP is involved in various mental health research activities in the context of COVID-19. MPi published two papers on mental health and the Covid-19 pandemic. This was unfunded. CC is the principal investigator for Co-SPACE, which is funded by UK Research and Innovation Council. RO'C reports grants from NIHR, grants from Medical Research Foundation, grants from Scottish Government, grants from NHS Health Scotland/Public Health Scotland, grants from Samaritans, grants from Scottish Association for Mental Health, grants from Mindstep Foundation, outside the submitted work; and he is co-chair of the Academic Advisory Group to the Scottish Government's National Suicide Prevention Leadership Group. He is also a member of the National Institute of Health and Care Excellence's guideline development group for the new NICE self-harm guidelines. PQ is involved in two COVID projects: the Covid-19, Social Media & Mental Health study (led by Dr. Margarita Panayiotou), and the Survey of Health and Wellbeing–Monitoring the Impact of COVID-19 (led by Dr. Michelle Lim, Iverson Health Innovation Research Institute, Swinburne University). The remaining authors declare that the research was conducted in the absence of any commercial or financial relationships that could be construed as a potential conflict of interest.

## Footnotes

^1^We define research infrastructure as the organizresources used by researchers to facilitate research, including research institutes, funding bodies, and research networks.

## References

[1] Holmes EA O'Connor RC Perry VH Tracey I Wessely S Arseneault L. Multidisciplinary research priorities for the COVID-19 pandemic: a call for action for mental health science. Lancet Psychiatry. (2020) 7:547–60. 10.1016/S2215-0366(20)30168-1 32304649 PMC7159850

[2] O'Connor DB Aggleton JP Chakrabarti B Cooper CL Creswell C Dunsmuir S. Research priorities for the COVID-19 pandemic and beyond: a call to action for psychological science. Br J Psychol. (2020) 111:1–27. 10.1111/bjop.12468 32683689 PMC7404603

[3] Mollica RF Cardozo BL Osofsky HJ Raphael B Ager A Salama P. Mental health in complex emergencies. Lancet. (2004) 364:2058–67. 10.1016/S0140-6736(04)17519-3 15582064

[4] National Institute for Health Research. RDS YH Public Involvement Fund. Available online at: https://www.rds-yh.nihr.ac.uk/public-involvement/rds-yh-public-involvement-fund/ (accessed July 9, 2020).

[5] University of Essex Institute for Social and Economic Research. Understanding Society: COVID-19 Study, 2020 [data collection]. (2020) Available online at: 10.5255/UKDA-SN-8644-3 (accessed August 1, 2020).

[6] Mental Health Research Matters. Mental Health Research Matters. Available online at: http://mentalhealthresearchmatters.org.uk (accessed July 9, 2020).

[7] Economic and Social Research Council. ESRC Application and Success Rate Data. Economic and Social Research Council (2017). Available online at: https://esrc.ukri.org/files/about-us/performance-information/application-and-success-rate-data-analysis-may-2018/ (accessed July 13, 2020).

[8] Wellcome Trust. Grant funding data 2018/19. London: Wellcome Trust (2020).

[9] Day NE. The silent majority: anuscript rejection and its impact on scholars. Acad Manag. 10:704–18. 10.5465/amle.2010.0027

[10] COVID-Minds. COVID-MINDS: Global Mental Health in the COVID-19 Pandemic. Available online at: https://www.covidminds.org (accessed July 9, 2020).

[11] National Institute for Health Research: Maudsley Biomedical Research Centre. COVID-19 and Mental Health Studies Register. Available online at: https://www.maudsleybrc.nihr.ac.uk/research/covid-19-studies/ (accessed July 9, 2020).

[12] Johnson R Grove A Clarke A. It's hard to play ball: a qualitative study of knowledge exchange and silo effects in public health. BMC Health Serv Res. (2018) 18:1–11. 10.1186/s12913-017-2770-6 29291745 PMC5748943

[13] Calderón-Larrañaga A Dekhtyar S Vetrano DL Bellander T Fratiglioni L. COVID-19: risk accumulation among biologically and socially vulnerable older populations. Ageing Res Rev. (2020) 63:101149. 10.1016/j.arr.2020.101149 32818650 PMC7430278

[14] Glasziou PP Sanders S Hoffmann T. Waste in covid-19 research. BMJ. (2020) 369:1–2. 10.1136/bmj.m1847 32398241

[15] Gunnell D Appleby L Arensman E Hawton K John A Kapur N. Suicide risk and prevention during the COVID-19 pandemic. Lancet Psychiatry. (2020) 7:468–71. 10.1016/S2215-0366(20)30171-1 32330430 PMC7173821

[16] National Institute for Health Research. INVOLVE: What is Public Involvement in Research. Available online at: http://www.invo.org.uk/find-out-more/what-is-public-involvement-in-research-2/ (accessed July 21, 2020).

[17] Paylor J McKevitt C. The possibilities and limits of “co-producing” Research. Front Sociol. (2019) 4:1–5. 10.3389/fsoc.2019.00023 33869348 PMC8022533

[18] Gillard S Dare C Hardy J Nyikavaranda P Olive RR Shah P. Experiences of living with mental health problems during the COVID-19 pandemic in the UK: a coproduced, participatory qualitative interview study. Soc Psychiatry Psychiatr Epidemiol. (2020):1–16. 10.1101/2020.11.03.20225169 33665680 PMC7931976

[19] Dewa LH Crandell C Choong E Jaques J Bottle A Kilkenny C. CCopeY: a mixed-methods coproduced study on the mental health status and coping strategies of young people during COVID-19 UK lockdown. J Adolesc Heal. (2021) 10.1016/j.jadohealth.2021.01.009 33589305 PMC9188746

[20] Hanley B Tarpey M. Involving the Public in COVID-19 Research: A Guest Blog by Bec Hanley and Maryrose Tarpey. Natl Heal Serv Heal Res Auth. (2020) Available online at: https://www.hra.nhs.uk/about-us/news-updates/involving-public-covid-19-research-guest-blog-bec-hanley-and-maryrose-tarpey/ (accessed July 21, 2020).

[21] Anna Freud National Centre for Children and Families. Young champions. Available online at: https://www.annafreud.org/on-my-mind/get-involved/young-champions/ (accessed July 21, 2020).

[22] Hickey G Richards T Sheehy J. Co-production from proposal to paper. Nature. (2018) 562:29–31. 10.1038/d41586-018-06861-9 30283117

[23] Murphy E Tierney E Ní Shé É Killilea M Donaghey C Daly A. COVID-19: Public and patient involvement, now more than ever. HRB Open Res. (2020) 3:35–44. 10.12688/hrbopenres.13067.1 32666039 PMC7327728

[24] Byrne L Wykes T. A role for lived experience mental health leadership in the age of Covid-19. J Ment Heal. (2020) 29:243–6. 10.1080/09638237.2020.1766002 32449392

[25] Gatera G. Why Lived Experience Matters. COVID Minds. (2020) Available online at: https://www.covidminds.org/post/why-lived-experience-matters-when-it-comes-to-mental-health-welfare-during-covid-19 (accessed August 31, 2020).

[26] Bootcamp C. Campaign Bootcamp. Available online at: https://campaignbootcamp.org (accessed July 21, 2020).

[27] Richards T Scowcroft H. Patient and public involvement in covid-19 policy making. BMJ. (2020) 370:m2575. 10.1136/bmj.m2575 32611571

[28] National Survivor User Network. COVID-19: Keeping in Touch With Each Other When We Can't Meet Face to Face. National Survivor User Network (2020). Available online at: https://www.nsun.org.uk/news/covid-19-keeping-in-touch-with-each-other-when-we-cant-meet-face-to-face (accessed September 4, 2020).

[29] Farr M Davies R Davies P Bagnall D Brangan E Andrews H. A Map of Resources for Co-producing Research in Health and Social Care. National Institute for Health Research ARC West and People in Health West of England; University of Bristol and University of West of England (2020). Available online at: https://arc-w.nihr.ac.uk/Wordpress/wp-content/uploads/2020/05/Map-of-resources-Web-version-v1.2.pdf (accessed September 4, 2020).

[30] National Institute for Health Research. INVOLVE: Guidance on Co-producing a Research Project. Southampton: National Institute for Health Research (2018).

[31] Rycroft-Malone J Burton CR Bucknall T Graham ID Hutchinson AM Stacey D. Collaboration and co-production of knowledge in healthcare: Opportunities and challenges. Int J Heal Policy Manag. (2016) 5:221–3. 10.15171/ijhpm.2016.08 27239867 PMC4818986

[32] National Institute for Health Research. Top Tips for Carring Out PPI Activities During COVID-19. (2020) Available online at: https://www.rds-sc.nihr.ac.uk/ppi-information-resources/ppi-covid19/ (accessed July 23, 2020).

[33] Demkowicz O Ashworth E. Teenagers' Experiences of Life in Lockdown (TELL). (2020) Available online at: https://www.maudsleybrc.nihr.ac.uk/research/covid-19-studies-project-details?id=9166 (accessed July 8, 2020).

[34] Short V. Experiences of Helpful Care Giving and Care Receiving in Mental Health Services During Covid 19 Lockdown. (2020) Available online at: https://www.maudsleybrc.nihr.ac.uk/research/covid-19-studies-project-details?id=9136 (accessed July 8, 2020).

[35] Midgley N. The Impact of COVID-19 and Lockdown on Foster Carers and Children in Care. (2020) Available online at: https://www.maudsleybrc.nihr.ac.uk/research/covid-19-studies-project-details?id=9140 (accessed July 8, 2020).

[36] Davidson L Ridgway P Kidd S Topor A Borg M. Using qualitative research to inform mental health policy. Can J Psychiatry. (2008) 53:137–44. 10.1177/070674370805300303 18441659

[37] The Lancet Psychiatry. Deeper understanding. Lancet Psychiatry. (2019) 6:713. 10.1016/S2215-0366(19)30304-9 31448749

[38] Greenhalgh T Annandale E Ashcroft R Barlow J Black N Bleakley A. An open letter to The BMJ editors on qualitative research. BMJ. (2016) 352:i957. 10.1136/bmj.i957 26865572

[39] Braun V Clarke V. Novel insights into patients' life-worlds: the value of qualitative research. Lancet Psychiatry. (2019) 6:720–1. 10.1016/S2215-0366(19)30296-2 31350207

[40] Neyman J. On the two different aspects of the representative method: The method of stratified sampling and the method of purposive selection. J R Stat Soc. (1934) 97:558–625. 10.2307/2342192

[41] Baker R Brick JM Bates NA Battaglia M Couper MP Dever JA. Report of the AAPOR task force on non-probability sampling. American Association for Public Opinion Research (2013). Available online at: https://www.aapor.org/AAPOR_Main/media/MainSiteFiles/NPS_TF_Report_Final_7_revised_FNL_6_22_13.pdf (accessed July 24, 2020).

[42] Cornesse C Blom AG Dutwin D Krosnick JA De Leeuw ED Legleye S. A review of conceptual approaches and empirical evidence on probability and nonprobability sample survey research. J Surv Stat Methodol. (2020) 8:4–36. 10.1093/jssam/smz041

[43] Pierce M Hope H Ford T Hatch S Hotopf M John A. Mental health before and during the COVID-19 pandemic: a longitudinal probability sample survey of the UK population. Lancet Psychiatry. (2020) 7:883–92. 10.2139/ssrn.3624264 32707037 PMC7373389

[44] Hartshorne TS. Psychometric properties and confirmatory factor analysis of the UCLA Loneliness Scale. J Pers. (1993) 61:182–95. 10.1207/s15327752jpa6101_14 16370798

[45] Ransing R Ramalho R Orsolini L Adiukwu F Gonzalez-Diaz JM Larnaout A. Can COVID-19 related mental health issues be measured? Assessment options for mental health professionals. Brain Behav Immun. (2020) 88:32–4. 10.1016/j.bbi.2020.05.049 32470593 PMC7248629

[46] Vogt DS King DW King LA. Focus groups in psychological assessment: Enhancing content validity by consulting members of the target population. Psychol Assess. (2004) 16:231–43. 10.1037/1040-3590.16.3.231 15456379

[47] Detmar SB Bruil J Ravens-Sieberer U Gosch A Bisegger C. The use of focus groups in the development of the KIDSCREEN HRQL questionnaire. Qual Life Res. (2006) 15:1345–53. 10.1007/s11136-006-0022-z 16826436

[48] Office for National Statistics. Deaths involving COVID-19, England and Wales: Deaths Occurring in March 2020. Office for National Statistics (2020). Available online at: https://www.ons.gov.uk/peoplepopulationandcommunity/birthsdeathsandmarriages/deaths/bulletins/deathsinvolvingcovid19englandandwales/deathsoccurringinmarch2020 (accessed July 13, 2020).

[49] Office for National Statistics. Coronavirus (COVID-19) Related Deaths by Ethnic Group, England and Wales: 2 March 2020 to 10 April 2020. Office for National Statistics (2020). Available online at: https://www.ons.gov.uk/peoplepopulationandcommunity/birthsdeathsandmarriages/deaths/articles/coronavirusrelateddeathsbyethnicgroupenglandandwales/2march2020to10april2020 (accessed July 13, 2020).

[50] Parkerson HA Thibodeau MA Brandt CP Zvolensky MJ Asmundson GJG. Cultural-based biases of the GAD-7. J Anxiety Disord. (2015) 31:38–42. 10.1016/j.janxdis.2015.01.005 25725310

[51] Burton J Lynn P Benzeval M. How understanding society: the UK household longitudinal study adapted to the COVID-19 pandemic. Surv Res Methods. (2020) 14:235–9. 10.18148/srm/2020.v14i2.7746

[52] Patalay P Deighton J Fonagy P Wolpert M. Equivalence of paper and computer formats of a child self-report mental health measure. Eur J Psychol Assess. (2015) 31:54–61. 10.1027/1015-5759/a000206

[53] Understanding Society. Main Survey User Guide (2020). Available online at: https://www.understandingsociety.ac.uk/sites/default/files/downloads/documentation/mainstage/user-guides/mainstage-user-guide.pdf (accessed July 24, 2020).

[54] Janszky I Ahlbom A Svensson AC. The Janus face of statistical adjustment: Confounders versus colliders. Eur J Epidemiol. (2010) 25:361–3. 10.1007/s10654-010-9462-4 20449636

[55] Foster EM. Causal inference and developmental psychology. Dev Psychol. (2010) 46:1454–80. 10.1037/a0020204 20677855

[56] Marlies M Nelemans SA Danneel S Fernández-Castilla B Van den Noortgate W Goossens L. Loneliness and social anxiety across childhood and adolescence: multilevel meta-analyses of cross-sectional and longitudinal associations. Dev Psychol. (2019) 55:1548–65. 10.1037/dev0000719 30896228

[57] Danneel S Bijttebier P Bastin M Colpin H Van den Noortgate W Van Leeuwen K. Loneliness, social anxiety, and depressive symptoms in adolescence: examining their distinctiveness through factor analysis. J Child Fam Stud. (2019) 28:1326–36. 10.1007/s10826-019-01354-3

[58] Demkowicz O Ashworth E O'Neill A Hanley T Pert K. Teenagers' experiences of Life in Lockdown: Briefing Two. The University of Manchester and Liverpool John Moores University (2020). Available online at: https://documents.manchester.ac.uk/display.aspx?DocID=50543 (accessed September 7, 2020).

[59] Hamaker EL Kuiper RM Grasman RPPP. A critique of the cross-lagged panel model. Psychol Methods. (2015) 20:102–16. 10.1037/a0038889 25822208

[60] Wellcome Trust. Sharing Research Data and Findings Relevant to the Novel Coronavirus (COVID-19) Outbreak. (2020) Available online at: https://wellcome.ac.uk/coronavirus-covid-19/open-data (accessed July 9, 2020).

[61] Cartwright-Hatton S Dodd H Lester K Banerjee R Gibson J Hurding R. Play First: Supporting Children's Social and Emotional Wellbeing During and After Lockdown. Available online at: https://www.sussex.ac.uk/about/documents/play-first–supporting-childrens-social-and-emotional-wellbeing-during-and-after-lockdown.pdf (accessed July 10, 2020).

[62] Fraser N Brierley L Dey G Polka JK Pálfy M Coates JA. Preprinting a pandemic: the role of preprints in the COVID-19 pandemic. bioRxiv [preprint]. (2020) 10.1101/2020.05.22.111294

[63] Nosek BA Ebersole CR DeHaven AC Mellor DT. The preregistration revolution. Proc Natl Acad Sci U S A. (2018) 115:2600–6. 10.1073/pnas.1708274114 29531091 PMC5856500

[64] Satinsky E Crepaz-keay D Kousoulis AA. Mental health service users' perceptions of data sharing and data protection: a short qualitative report. J Innov Heal Informatics. (2018) 25:239–42. 10.14236/jhi.v25i4.1033 30672404

[65] Aitken M De St Jorre J Pagliari C Jepson R Cunningham-Burley S. Public responses to the sharing and linkage of health data for research purposes: a systematic review and thematic synthesis of qualitative studies. BMC Med Ethics. (2016) 17:1–24. 10.1186/s12910-016-0153-x 27832780 PMC5103425

[66] Megoran N Mason O. Second Class Academic Citizens: The Dehumanising Effects of Casualisation in Higher Education. London: UCU (2020)

[67] Byrom N. COVID-19 and the research community: The challenges of lockdown for early-career researchers. eLife Sci. (2020) 9:e59634. 10.7554/eLife.59634 32530421 PMC7292644

[68] Levine RL Rathmell WK. COVID-19 impact on early career investigators: a call for action. Nat Rev Cancer. (2020) 20:357–8. 10.1038/s41568-020-0279-5 32503987 PMC7273376

[69] Paula JR. Lockdowns due to COVID-19 threaten PhD students' and early-career researchers' careers. Nat Ecol Evol. (2020) 4:999. 10.1038/s41559-020-1231-5 32493949 PMC7392882

[70] Yan W. Early-Career Scientists at Critical Career Junctures Brace for Impact of COVID-19. ScienceMag. (2020) Available online at: https://www.sciencemag.org/careers/2020/04/early-career-scientists-critical-career-junctures-brace-impact-covid-19 (accessed July 13, 2020).

[71] Staniscuaski F Reichert F Werneck FP de Oliveira L Mello-Carpes PB Soletti RC. Impact of COVID-19 on academic mothers. Science (80-). (2020) 368:724. 10.1126/science.abc2740 32409466

[72] Viglione G. Are women publishing less during the pandemic? Here's what the data say. Nature. (2020) 581:365–6. 10.1038/d41586-020-01294-9 32433639

[73] World Health Organization. Substantial Investment Needed to Avert Mental Health Crisis (2020) Available at: https://www.who.int/news-room/detail/14-05-2020-substantial-investment-needed-to-avert-mental-health-crisis (accessed July 21, 2020).

[74] May R Peetz D Strachan G. The casual academic workforce and labour market segmentation in Australia. Labour Ind a J Soc Econ relations Work. (2013) 23:258–75. 10.1080/10301763.2013.839085

[75] University and College Union. Precarious Work in Higher Education Insecure Contracts and how they have Changed Over Time. London (2020) 19:1–61.

[76] O'Keefe T Courtois A. ‘Not one of the family': Gender and precarious work in the neoliberal university. Gender, Work Organ. (2019) 26:463–79. 10.1111/gwao.12346

[77] Milojevic S Radicchi F John P. Changing demographics of scientific careers: The rise of the temporary workforce. Proc Natl Acad Sci U S A. (2019) 116:12616–23. 10.1073/pnas.1800478115 30530691 PMC6294951

[78] Han RH Schmidt MN Waits WM Bell AKC Miller TL. Planning for mental health needs during COVID-19. Curr Psychiatry Rep. (2020) 22:66. 10.1007/s11920-020-01189-6 33030637 PMC7542088

[79] UK Research and Innovation. Guidance for the Research and Innovation Communities. (2020) Available online at: https://www.ukri.org/research/coronavirus/guidance-for-the-research-and-innovation-communities1/ (accessed July 23, 2020).

[80] Wellcome Trust. Coronavirus (COVID-19): Information for Grant Applicants and Grantholders. (2020) Available online at: https://wellcome.ac.uk/grant-funding/guidance/coronavirus-covid-19-information-grant-applicants-and-grantholders (accessed July 24, 2020).

[81] National Institute for Health Research. NIHR Launches New UK Wide Funding Call for Longer-Term COVID-19 Research. (2020) Available online at: https://www.nihr.ac.uk/news/nihr-launches-new-uk-wide-funding-call-for-longer-term-covid-19-research/25013 (accessed July 21, 2020).

[82] UKRI. MRC/AHRC/ESRC Adolescents, Mental Health and the Developing Mind: Engagement Awards. Available online at: https://mrc.ukri.org/research/initiatives/adolescence-mental-health-and-the-developing-mind/ (accessed July 9, 2020).

[83] UK Government. Government Launches Plan to Tackle Loneliness During Coronavirus Lockdown. UK Government (2020). Available online at: https://www.gov.uk/government/news/government-launches-plan-to-tackle-loneliness-during-coronavirus-lockdown (accessed February 7, 2021).

